# A case of rectal cancer complicated with segmental arterial mediolysis (SAM) safely treated with curative resection - A case report

**DOI:** 10.1016/j.ijscr.2024.109418

**Published:** 2024-02-20

**Authors:** Tamuro Hayama, Hiroki Ochiai, Keijiro Nozawa, Yoshinao Kikuchi, Yuko Sasajima, Takeo Fukagawa

**Affiliations:** a Department of Surgery, Teikyo University School of Medicine, Japan; b Department of Pathology, Teikyo University School of Medicine, Japan

**Keywords:** Colorectal cancer, Segmental arterial mediolysis, Surgery, Case report

## Abstract

**Introduction:**

Recent advances in diagnostic imaging techniques have led to an increasing number of case reports of segmental arterial mediolysis (SAM). However, reports of abnormalities associated with SAM of abdominal organs, including the bowel, are limited. SAM, a rare vascular disease that causes spontaneous intra-abdominal bleeding, including shock and intestinal ischemia, has been reported to be associated with high mortality, but it has not been reported to coexist with rectal cancer.

**Case presentation:**

A 74 year-old male was referred to our hospital with a rectal cancer and he was admitted for further examination. Computed tomography angiography (CTA) revealed dissection and aneurysm in the celiac artery, superior mesenteric artery (SMA), and the inferior mesenteric artery were dilated, leading to a diagnosis of SAM.

**Clinical discussion:**

Surgery for rectal cancer requires cutting the inferior mesenteric artery. The risk of bleeding during surgery increases when SAM is associated with the inferior mesenteric artery. The radical surgery for rectal cancer was executed without complications, including significant bleeding. This was achieved through careful management of SAM, meticulous control of blood pressure throughout the surgical procedure, and the delicate treatment of the SMA. A pathological diagnosis of the resected inferior mesenteric artery at the time of radical surgery was performed, and a definitive diagnosis of SAM was made.

**Conclusion:**

We present a first known case in which high anterior resection was successfully performed for rectal cancer complicated by SAM. The relationship between cancer and SAM is unclear and further case accumulation is needed.

## 1 Introduction

Segmental arterial mediolysis (SAM) is a rare, non-inflammatory, non-atherosclerotic disease of unknown etiology characterized by degeneration of the affected vascular mid-layer. Degeneration of the lining of blood vessels causes susceptibility to vessel dissection, hemorrhage and ischemia [1,2]. SAM was first reported by Slavin in 1976 [1]. The exponential increase in the use of computed tomography (CT) and magnetic resonance imaging (MRI) has led to early diagnosis of asymptomatic patients with SAM. This report describes a case of rectal cancer diagnosed with SAM by preoperative three-dimensional computed tomography angiography (CTA).

## 2 Case presentation

**Fig. 1:**
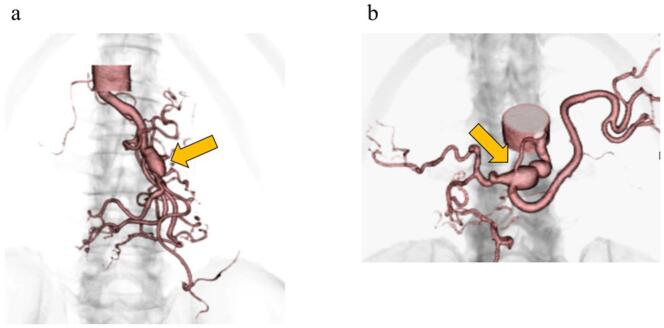
Abdominal computed tomography angiography scan. a: CTA of the abdomen: Pseudoaneurysm of superior mesenteric artery observed by three-dimensional computed tomography angiography (arrow). b: CTA of the abdomen shows pseudoaneurysm (arrow) of the celiac artery.

**Fig. 2:**
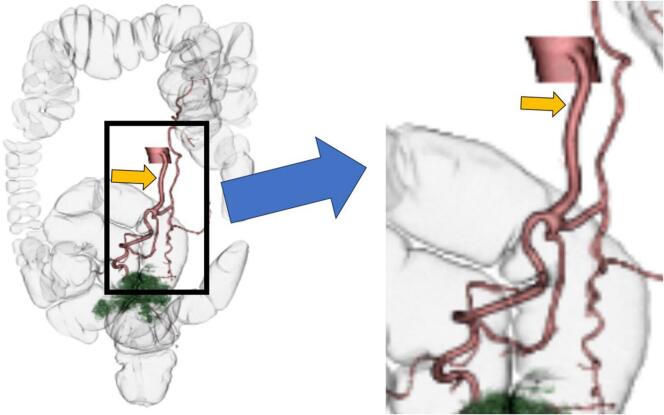
Virtual enema image and Abdominal computed tomography angiography scan. The green part represents the tumor, and the inferior mesenteric artery expands from the middle and an aneurysm is recognized (arrow). (For interpretation of the references to color in this figure legend, the reader is referred to the web version of this article.)

A 74-year-old man without a significant medical history was diagnosed with rectal cancer at a local clinic and was referred to our hospital for further work-up and treatment of rectal cancer. There was no history of previous illnesses. Abdominal examination revealed normal findings, and the laboratory data indicated no abnormal values. Colonoscopy findings revealed a 60-mm type 2 lesion observed in the RS. He was diagnosed with advanced rectal adenocarcinoma with lymph node metastasis (N1) and no distant metastasis (M0). A preoperative three-dimensional CTA scan incidentally revealed dissection and aneurysm in the celiac artery and superior mesenteric artery, and the inferior mesenteric artery was dilated, leading to a diagnosis of SAM (Figs. 1, 2). In recent years, it has been reported that asymptomatic SAM has been treated conservatively and is doing well [3,4]. Preoperative examination revealed that the SMA (superior mesenteric artery) was complicated by an aneurysm. The patient was finally diagnosed with rectal cancer with SAM (T4aN1M0, Stage III). The feeding artery for rectal cancer is the IMA, and the IMA must also be ligated when rectal cancer is resected.

**Fig. 3:**
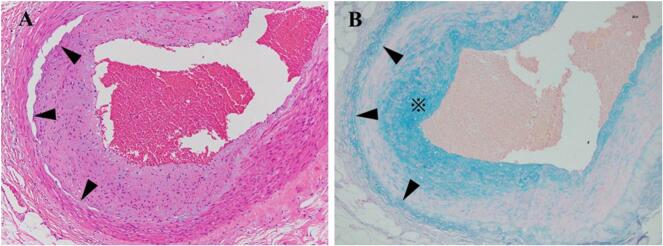
A. Disruption of the smooth muscle in the media (arrowheads) with associated mucoid intimal changes. (H&E, 400×). B. Mucous staining reveals mucosal degeneration in the intima (*) and between the media and adventitia (arrowhead) (alcian blue staining, 400×). (For interpretation of the references to color in this figure legend, the reader is referred to the web version of this article.)

**Fig. 4:**
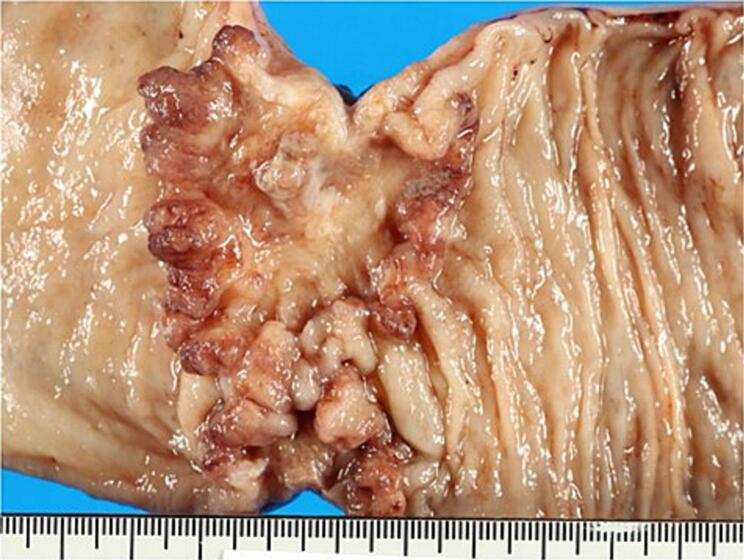
Pathological specimen. A type 2 lesion is found in the center of the specimen.

Recognizing the concurrent presence of SAM in the celiac artery, SMA, and IMA, it was concluded that there existed a heightened risk of intraoperative bleeding. Subsequent to obtaining informed consent from both the patient and their family, a radical surgery was scheduled. High anterior resection was performed with the external approach to mobilize the sigmoid colon and rectum. In this case, SAM decided to be treated conservatively, without interventional radiology and surgery. Blood pressure was controlled below 150 mmHg during surgery. We confirmed the color tone of the small intestine and confirmed that there was no decrease in intestinal blood flow due to dissection of the SMA. Movement of the small bowel was performed carefully to avoid aneurysm rupture caused by injury. The operative time was 184 min, intraoperative blood loss was 180 ml, and no immediate or late complications including intra-abdominal hemorrhage were observed. Pathological examination was performed on resected IMA and rectal cancer. IMA shows evidence of mucodegeneration of the media, cleft formation between the media and adventitia, and mucosal degeneration between the media and adventitia on mucus staining (Fig. 3). Based on the above, SAM was diagnosed. The resected specimen found an ulcerative type 2 tumor measuring 67 × 45 mm (Fig. 4). The pathological examination revealed that the type2 tumor invaded beyond the serosa layer (T4a). We identified 28 lymph nodes harvested, it did reveal one lymph node metastasis. Subsequently, the patient was classified as having Stage III (T4aN1M0) rectal cancer. The postoperative clinical course was favorable, and the patient was discharged on the 13th day after surgery. The patient did not wish to receive adjuvant chemotherapy. The patient remained stable and healthy, with no evidence of rectal cancer recurrence, eight months after surgery. In addition, there were no symptoms of SAM and no intra-abdominal bleeding such as aneurysm rupture was observed.

This work has been reported in line with the SCARE criteria [5].

## 3 Discussion

In recent years, with the development of diagnostic imaging, SAM has become widely recognized [6]. Although the relationship between cancer and SMA is unclear, there are no case reports of rectal cancer with SAM. It was reported that the most common abdominal artery associated with SAM (143 cases) was the superior mesenteric artery (53 %), followed by hepatic artery (45 %), celiac artery (36 %), renal artery (26 %), and the splenic artery (25 %) [7]. We searched for the keywords “Segmental arterial mediolysis” and “colorectal cancer” in PubMed found no case. Moreover, we searched for the keywords “Segmental arterial mediolysis” and “rectal” found one case. This case was the first known reported case of intra-abdominal hemorrhage with segmental arterial mediolysis requiring emergent hemicolectomy [8].

The gold standard for diagnosis of SAM is a pathological finding involving injurious and reparative phases in the arterial lesions of the surgical specimens [1,2]. These injurious states include mediolysis, formation of arterial gaps, and separation of the outer media, there is no evidence of inflammation and ischemia [9,10]. However, with the rapid progress of CTA, the number of cases diagnosed as SAM is increasing as a result of a combination of diagnostic imaging and clinical criteria [7]. Our case was diagnosed as SAM based on combined imaging and pathologic findings.

Clinical manifestations of SAM range from abdominal pain to acute intra-abdominal hemorrhage or organ ischemia [11]. Although the prognosis remains unclear, abdominal visceral aneurysm rupture has a poor prognosis, and the mortality rate is reported to be 25 % for splenic artery rupture, 35 % for hepatic artery rupture, and 50 % for pancreaticoduodenal artery rupture [12]. Regarding prognosis, a median follow-up period of 3 years using CTA has been reported. That report indicated that segmental arterial mediolysis lesions may resolve or remain unchanged in SMA [13] [14]. The relationship between SAM and rectal cancer is unclear, and it will be important to accumulate more cases in the future. The feeding artery for rectal cancer is IMA, resection of the IMA is required for curative resection for rectal cancer. The tissue of IMA combined with SAM is extremely fragile, and caution is required as the blood vessels may rupture at any time. We were able to maintain intraoperative blood pressure below 150 mmHg and treat IMA gently, and safely performed the surgery. The mortality rate associated with open surgery for SAM is documented at 25 %. However, there have been no reported instances of bleeding from ligated and dissected SAM vessels [6]. Since there has been no report of SAM complication in the arteries feeding colorectal cancer, further accumulation of cases is necessary.

This case was diagnosed with SAM by 3D CTA performed before surgery for rectal cancer. He underwent radical surgery for rectal cancer and was discharged without postoperative complications. We report a first case of successful rectal cancer surgery complicated with SAM.

## Consent for publication

Written informed consent was obtained from the patient for publication and any accompanying images. A copy of the written consent is available for review by the Editor-in-Chief of this journal on request.

## Ethical approval

Ethical approval for this study (Ethical Committee 16-032) was provided by the Ethical Committee Teikyo University, Tokyo, Japan on 23 August 2016.

## Funding

This work was supported by JSPS KAKENHI Grant Number JP 22K08784 and ACRO Research Grants of Teikyo University.

## Author contribution

Surgical assistance: TH, KN; manuscript preparation: HO; supervision: YK, TF and YS. All the authors read and approved the final manuscript.

## Guarantor

Tamuro Hayama.

## Conflict of interest statement

The authors declare no conflicts of interest associated with this manuscript.

## Data Availability

All available data supporting our findings are listed in the tables which are shown in the manuscript. Thus, our conclusions can be ascertained from the published data.
